# Long-term a posteriori dietary patterns and risk of hip fractures in a cohort of women

**DOI:** 10.1007/s10654-017-0267-6

**Published:** 2017-06-05

**Authors:** Eva Warensjö Lemming, Liisa Byberg, Håkan Melhus, Alicja Wolk, Karl Michaëlsson

**Affiliations:** 1 Department of Surgical Sciences, Section of Orthopedics, Uppsala University, Dag Hammarskjölds väg 14B, UCR/MTC, Uppsala Science Park, 751 83 Uppsala, Sweden; 2 Department of Medical Sciences, Clinical Pharmacology, Uppsala University, Uppsala, Sweden; 3 Division of Nutritional Epidemiology, Institute of Environmental Medicine, Karolinska Institutet, Stockholm, Sweden

**Keywords:** Dietary pattern, Healthy dietary pattern, Western dietary pattern, Principal component analysis, Hip fractures, Food frequency questionnaire

## Abstract

Dietary pattern analysis is a useful tool to study the importance of food components in the context of a diet and how they relate to health and disease. The association between dietary patterns and fractures is at present uncertain. We aimed to study associations between dietary patterns and risk of hip fracture in the Swedish Mammography Cohort, including 56,736 women (median baseline age 52 years). Diet data was collected in food frequency questionnaires at two investigations and dietary patterns were defined by principal component analysis using 31 food groups. Information on hip fractures was collected from the Swedish National Patient Register. Multivariable adjusted hazard ratios (HR) with 95% confidence intervals (CI) were estimated in Cox proportional hazards regression analysis. The two patterns identified—the healthy and Western/convenience dietary patterns—were time-updated and analysed. During a median follow-up time of 25.5 years, 4997 women experienced a hip fracture. Hip fracture rate was 31% lower in the highest compared to the lowest quartile of the healthy dietary pattern [HR (95% CI) 0.69 (0.64; 0.75)]. In contrast, women in the highest compared to the lowest quartile of the Western/convenience dietary pattern had a 50% higher [HR (95% CI) 1.50 (1.38; 1.62)] hip fracture rate. Further, in each stratum of a Western/convenience dietary pattern a higher adherence to a healthy dietary pattern was associated with less hip fractures. The present results suggest that a varied healthy diet may be beneficial for the prevention of fragility fractures in women.

**Electronic supplementary material:**

The online version of this article (doi:10.1007/s10654-017-0267-6) contains supplementary material, which is available to authorized users.

## Introduction

Every year millions of people throughout the world suffer from a fragility fracture [1] and these fractures are associated with loss of quality of life [2] increased mortality [3] and high health care costs [2]. To reduce the burden from fractures in society, it is important to explore preventive strategies that may impact risk and are modifiable by intervention. One such modifiable lifestyle factor is diet. Much research relating to diet and bone health has historically been spent on calcium and vitamin D [4, 5]. However, many other nutrients and food components in our diets, as well as individual foods may influence bone health [2, 4, 6–10]. The influence of individual components on bone health and fractures is weak to moderate and evidence inconclusive. In addition, all nutrients and other bioactive compounds are consumed as foods in the context of a diet and the food matrix may deliver effects beyond the sum of its components [11, 12].

Dietary patterns in a population can be formed either using a posteriori data driven methods, such as principal component analysis (PCA) or cluster analysis or a priori forming indices (diet scores), both with strengths and pitfalls [13]. PCA produces continuous dietary pattern variables reflective of actual reported dietary intakes and do not necessarily represent the most healthy or unhealthy pattern. These continuous variables are easily applied in regression models and appear to be advantageous compared to clusters identified in cluster analysis [14]. Previous research on the link between dietary patterns and risk of fractures has generated inconsistent results and studies that have used PCA or factor analysis are few [15–19]. Using a posteriori data driven diet scores, a study from the US reported no association between neither a prudent nor a Western dietary pattern and risk of hip fractures [16], while a nutrient dense pattern was inversely related to low trauma fractures in Canada [17]. The a priori defined Mediterranean diet score, reflective of a diet high in plant foods, unsaturated fatty acids, fish, nuts and whole grains, was related to a decreased risk of hip fracture in US women [20] as well as in a previous study in the present cohort of women and in Swedish men [21]. Also, when studying individual foods as the exposure, higher intakes of vegetables and fruits were related to a lower risk of hip fracture in different settings [6, 22] and a higher consumption of soda was related to a greater risk of hip fracture in women in the US [23].

Different dietary patterns may thus differentially influence the rate of hip fracture, the most devastating type of fragility fracture. The aim of our study was to examine the strength and direction of associations between dietary patterns defined a posteriori by PCA and hip fracture in a large cohort of Swedish women.

## Methods

### Study cohort

**Fig. 1:**
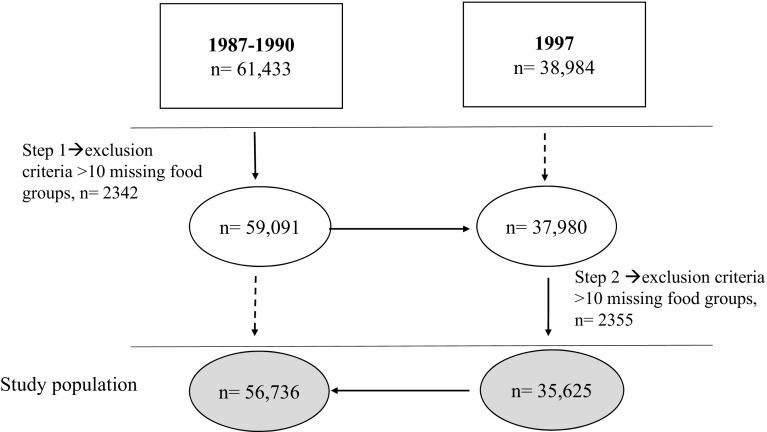
The study sample was formed with data from the two food frequency questionnaires (FFQ) in a two-step approach excluding those participants with missing data on ≥10 food groups in each step. In the first step (step 1), the 31 food groups were formed with the data from the 1987-FFQ (n = 61,433) leaving 59,091 participants. In the second step (step 2), the 31 food groups were formed with data from the 1997-FFQ (n = 38,984) in those individuals remaining after the exclusions in step 1 (n = 37,980). This left 35,625 individuals from the 1997 investigation and yielded 56,736 participants to follow-up from baseline in 1987–1990

The Swedish Mammography Cohort (SMC) was established in 1987–1990. All 90,303 women born between 1914 and 1948 and residing in two Swedish counties, were invited to a mammography screening. Enclosed with this invitation was a questionnaire covering diet and lifestyle, which was completed by 74% of the women. Subjects were excluded if the national registration number was missing, the questionnaire had not been dated or energy intakes were deemed as implausible (±3 SD from the mean value of the ln-transformed energy intake). Subjects with prior cancer diagnosis, except those with non-melanoma skin cancer were also excluded. In late 1997 a second expanded questionnaire was sent to those still living in the study area (response rate 70%). The cohort with exclusions has been described previously [24] and consists of 61,433 (baseline) and 38,984 (1997) women. For the present analysis we defined our study sample based on food groups generated with data from the two FFQs in a two-step approach. We excluded individuals with more than 10 of the food groups missing at each investigation as described in Fig. 1, to avoid the poorest assessments but careful not to introduce collider bias by for example influences from socio-economic status [25, 26]. This left 56,736 (baseline) and 35,625 (1997) women for the present analysis. Any remaining missing data on food groups was considered as zero consumption. The study was approved by the regional ethics committees at Uppsala University, Uppsala, and Karolinska Institutet, Stockholm, Sweden.

### Dietary assessment and food groupings

The dietary assessment [24] (http://ki.se/en/imm/the-swedish-mammography-cohort-smc) consisted of food-frequency questionnaires (FFQs) that included 67 and 96 food items in 1987 and 1997, respectively. Participants indicated in the FFQs how often, on average, they had consumed each food item during the past 6 months (1987 FFQ) or the last year (1997 FFQ) and chose from 8 predefined frequency categories ranging from “never/seldom” to “4 or more times per day” (1987 FFQ) or “3 or more times per day” (1997 FFQ). Frequently consumed foods such as dairy products and bread were additionally reported as number of servings per day. Energy and nutrient intakes were estimated by multiplying the consumption frequency of each food item with the nutrient content of age specific portion sizes. Nutrient values were obtained from the Swedish food composition database, National Food Agency. Nutrient intakes were adjusted for total energy intake using the residual method [27].

### Food groups

Frequency data expressed as the consumption frequency per day were collapsed into 31 food groups, based on similarities in nutrients and culinary use. Some food groups consisted of just one food or beverage, for example tea, coffee, eggs and strong beer because they did not fit into any other group or were considered a food group on their own. A previous study conducted in SMC indicated differential effects of fermented milk and non-fermented milk (described onwards as milk) on fracture risk [8]. Therefore, separate milk and fermented milk food groups were formed. Further, since the 1997 FFQ asked about a greater number of food items, partly due to the changing food market, a food group will consequently reflect information from a greater number of food items in 1997 compared to 1987, as indicated in Table 1.

**Table 1:** Component loadings for the healthy and Western/convenience dietary patterns defined by principal component analysis at baseline and 1997

Food groups	Baseline 1987–1990, n = 56,736			1997, n = 35,625		
	Description	1. Healthy	2. Western/convenience	Description	2. Healthy	3. Western/convenience
Vegetables	Cabbage, tomatoes, spinach and kale, lettuce, cucumber, root vegetables, legume dish	0.4306		Cabbage, tomatoes, spinach, lettuce, cauliflower, broccoli and Brussel sprouts, cucumber, carrots, beet root, legume dish, onion, garlic, mixed vegetables, soya bean products	0.3844	
Fruits	Apples and pear, citrus fruits, banana, fruit juice	0.3704		Apples, pears, citrus fruits, banana, berries, other fruits, fruit juice	0.4383	
Fried potatoes	Grilled and fried potatoes, French fries		0.2900	Grilled and fried potatoes, French fries	−0.1528	
Boiled potatoes			0.1683	Boiled potatoes		0.1542
Cereals	Porridge, gruels and breakfast cereals	0.2600		Porridge, gruels and breakfast cereals	0.3686	
Rice and pasta		0.1800		Rice and pasta		
Crisp bread	Swedish rye crisp bread			Swedish rye crisp bread	0.1773	0.2410
Whole meal bread		0.2528		Whole meal bread	0.3628	
White bread		−0.1582	0.3452	White bread		0.3071
Meat	Meat, meat stew, sausages, bacon, ham, minced meat, cold cuts		0.3734	Minced meat, pork, beef and veal, sausage and cold cuts		
Offals	Black pudding, liver pâté, liver and kidney		0.2220	Black pudding, liver pâté, liver and kidney foods		
Poultry		0.2385				−0.1876
Fatty fish	Herring, mackerel, salmon	0.2942		Herring, mackerel, salmon		
Other fish and shell fish	Other fish and shellfish (shrimp, mussels, crab)	0.3390		Cod, saithe, fish fingers and shellfish (shrimp, crayfish)		
Eggs	Eggs and scrambled egg	0.2119	0.1631	Eggs and omelette		
Milk	All fat types		0.1810	All fat types		0.2586
Fermented milk	Soured milk and yoghurt, all fat types, plain and flavoured	0.2803		Soured milk and yoghurt, all fat types, plain and flavoured	0.3123	
Cheese	All fat types		0.1640	Cheeses, cottage cheese, quark-all fat types	0.2516	0.2149
Margarine	Spread on bread			*High Fat dairy*-Cream and sour cream		0.2282
Butter	Spread on bread		0.1472	*Vegetable fats*-Dressings and mayonnaise	0.1709	
Soda	Soda and cordials		0.2811	Sodas		0.2493
Wine and spirits				Wine and spirits		
Light beer	Alcohol content, 0.5–2.8%			Alcohol content, 0.5–2.8%		
Beer	Alcohol content, >4.5%			Alcohol content, >4.5%		
Coffee				Coffee		0.2048
Tea				Tea	0.1590	
Convenience foods	Pancakes and waffles		0.2083	Pancakes, waffles, pizza, ketchup		0.1570
Sugary items	Sugar, jams and marmalades, sweet soups		0.3039	Sugar and honey, jams and marmalades, sweet soups		0.3591
Sweet bakery products and ice cream	Ice cream, cakes, cookies and pastries		0.2905	Ice cream, cakes, cookies and pastries		0.4097
Candy and chocolates			0.2437	Candy and chocolate		0.3046
Savoury snacks	Chips, popcorn, cheese doodles and nuts		0.1598	Chips, popcorn, cheese doodles and nuts		
Proportion of variance %		7.4	7.2		5.9	5.4
Eigenvalues		2.28	2.24		1.8	1.7

The factors are orthogonally rotated and loadings ±0.15 are shown

### Hip fracture ascertainment

We considered outcomes registered between study baseline (1987–1990) and 31 December 2014. Hip fracture cases were defined by the ICD10 codes S720, S721 and S722. Information on incident hip fracture events was obtained through individual linkage to the Swedish National Patient Registry [28] and by the use of a previously validated and accurate method [29]. The matching was almost complete because of the unique identification number assigned to all Swedish permanent residents.

### Covariates

Covariates were chosen using directed acyclic graphs (DAG) [30]. The covariates obtained from the questionnaires were use of calcium and multivitamin supplements, weight (kg) and height (m), smoking habits, living alone, menopausal status, educational level and leisure time physical activity during the past year. Body Mass Index (BMI) was calculated as weight (kg) divided by height squared (m^2^). The physical activity question had five pre-defined levels ranging from 1 h to more than 5 h per week and this question has been found valid compared to activity records and accelerometer data [31]. Comorbidity, expressed as Charlson’s weighted comorbidity index [32, 33] was defined by ICD diagnosis codes (versions 8, 9 and 10) from the National Patient Register before baseline and 1 January 1998. Hip fractures that occurred before baseline were indicated as previous fractures. Information on the frequency of falls before baseline and between the two investigations was retrieved from the Swedish National Patient Registry.

### Statistical analysis

Dietary pattern components were formed in exploratory PCA using 31 food groups expressed as the consumption frequency per day, at each investigation separately. Which components to retain from the analysis was primarily based on the Scree plot, eigenvalues (>1.5) and interpretability of the components. The PCA was then repeated with the number of components to retain and components were varimax rotated to become orthogonal (uncorrelated factors). The dietary patterns were interpreted and named on the basis of the components loadings (Table 1). The loadings for each pattern were obtained as a weighted sum of the reported frequencies of food groups and their loadings and each participant was assigned a score for each dietary pattern. To be considered in the Cox-analysis, the dietary pattern had to be formed at both investigations since quartiles of the time-updated dietary patterns were used, using information from both investigations [34]. Correlations between dietary patterns formed at baseline and 1997 as well as between dietary patterns and nutrients were investigated with Spearman rank correlations. For each participant follow-up time was accrued from baseline (primary analysis) or 1 January 1998 (sensitivity analysis) until date of hip fracture, date of death or the end of the study period (31 December 2014) using age as primary time scale. We estimated age and multivariable adjusted hazard ratios by Cox proportional hazards regression and 95% confidence intervals [CI] for the relation between quartiles of time-updated dietary patterns and hip fracture. The proportional hazard assumptions in the Cox models were confirmed graphically by comparing Nelson-Aalen plots and formally with Schoenfeld’s test. The main multivariable adjusted model included height (continuous), educational level (≤9, 10–12, >12 years, other), living alone (yes or no), calcium-supplement (yes or no), multivitamin-use (yes or no), physical activity level (5 categories), previous fractures (yes or no), menopausal-status (yes or no) and Charlson’s comorbidity index (continuous; 1–16) (Model I). In an additional analysis, fermented milk and milk were added to Model I in the analysis with the healthy and Western/convenience patterns, respectively. Further, energy intake, BMI (both continuous) and smoking status (current, former, never)—not primarily considered as confounders according to the DAG—were added to Model I and are referred to as Model II. Continuous covariates were time-updated and missing data on covariates were imputed using Stata’s “mi” package. We used 20 imputations to reduce sampling error. The multiple imputation takes into account model variables and produce 20 separate datasets. The Cox analysis is then run on all separate datasets and the results are combined. The variables living alone, use of calcium and multivitamin supplements, menopausal status, smoking and level of physical activity that were only available in the 1997 questionnaire were imputed, as well as educational level, BMI and height (less than 5% missing). In sensitivity analysis the Cox regression analysis was repeated using complete data, as well as using dietary patterns formed at each investigation. We additionally included history of falls as a covariate in the sensitivity analysis. Further, to study the impact of consistency or change in dietary patterns, the extreme quartiles of the Healthy dietary pattern at each investigation was used to create a three level categorical variable which was coded as [low 1987–low 1997] and set to the reference category, [low 1987–high 1997] and [high 1987–high 1997] and then used in the Cox regression. The baseline of this specific analysis was the second survey (January 1st, 1998). Lastly, study participants were jointly classified in nine strata across the tertiles of the time-updated Healthy and Western/convenience patterns and then used in the Cox regression. *P* values <0.05 were considered statistically significant. All analyses were carried out in Stata version 12.0 (Stata Corp., College Station, TX, USA).

## Results

### Dietary patterns

Four components were retained from each investigation on the basis of the scree plots, eigenvalues (<1.5) and interpretability of the components. The components explained 25% of the variance in the data at both investigations. The components were named based on the component loadings, which can be viewed as the correlation between the food group and the pattern. The patterns formed at baseline were named Healthy, Western/convenience, Alcohol & snack and Sandwich patterns. The patterns formed with 1997 data were named the Meat, grain and potatoes, Healthy, Western/convenience and Alcohol & snacks patterns. The Healthy pattern correlated between the investigations (rho = 0.48). The Western/convenience and the Alcohol & snack patterns were also correlated (rho = 0.41 for both) between investigations. The Sandwich (1987) and the Meat, grain and potatoes (1997) patterns were weakly correlated with other patterns and are therefore not retained for further analysis. The food group loadings and eigenvalues for the healthy and Western/convenience dietary patterns are presented in Table 1. At baseline, the Healthy pattern loaded high on fish (all types), cereals and whole meal bread, poultry, eggs, pasta and rice, fruits, vegetables and fermented milk, while the Western/convenience pattern was characterized by readily available food items such as sweet snacks and bakery products, sugar, jams, sodas and savoury snacks as well as meat and white bread. The Healthy pattern (1997) was similar to the Healthy pattern at baseline, but lacked higher loadings of fish and poultry. The Western/convenience pattern was also similar to that formed at baseline but also had loadings of high fat dairy (sour cream and cream) and lacked higher loadings from meat. The Alcohol & snack pattern loaded high on different alcoholic beverages as well as snack types of food, but were weakly related to hip fractures (Supplemental Table 1) and are therefore not further discussed in the present study.

### Baseline characteristics

Baseline characteristics in quartiles I and IV for the time-updated Healthy and Western/convenience patterns are presented in Table 2. Notably the energy intake and physical activity level was higher in the highest quartile of the Healthy and Western/convenience dietary pattern.

**Table 2:** Characteristics of study participants at baseline, 1987–1990, in quartile I and IV of respective time-updated dietary pattern; the Healthy and the Western/convenience patterns

Dietary pattern	Healthy		Western/convenience	
Quartile	I	IV	I	IV
Respective dietary pattern score	−1.7 ± 0.5	2.0 ± 1.2	−1.6 ± 0.5	2.0 ± 1.2
Number of participants	14, 184	14, 184	14, 184	14, 184
Number of hip fractures	1501	1002	1066	1414
Age, years (mean ± SD)	54 ± 10	52 ± 9	54 ± 9	53 ± 10
Body mass index, kg/m^2^ (mean ± SD)	24.9 ± 4.0	24.5 ± 3.9	24.9 ± 4.0	24.5 ± 4.0
Weight, kg (mean ± SD)	67 ± 11	67 ± 11	67 ± 11	66 ± 11
Height, m (mean ± SD)	1.64 ± 0.59	1.65 ± 0.57	1.64 ± 0.58	1.64 ± 0.59
Frequency per day (mean ± SD)				
Vegetables	1.0 ± 0.6	2.9 ± 1.6	1.9 ± 1.4	1.8 ± 1.2
Fruits	1.0 ± 0.8	2.7 ± 1.5	1.9 ± 1.3	1.7 ± 1.3
Cereals	0.3 ± 0.4	0.8 ± 0.6	0.6 ± 0.5	0.5 ± 0.5
Whole meal and crisp bread	1.5 ± 1.0	2.8 ± 1.3	1.9 ± 1.1	2.3 ± 1.3
Meat	0.7 ± 0.4	0.9 ± 0.6	0.5 ± 0.3	1.1 ± 0.6
Fish, including shellfish	0.1 ± 0.1	0.3 ± 0.3	0.2 ± 0.2	0.2 ± 0.2
Milk	0.8 ± 1.0	0.8 ± 1.0	0.5 ± 0.7	1.2 ± 1.1
Fermented milk and yoghurt	0.2 ± 0.3	0.8 ± 0.6	0.5 ± 0.6	0.4 ± 0.5
Cheese	1.0 ± 0.8	1.4 ± 0.9	0.9 ± 0.7	1.5 ± 1.0
Butter	0.7 ± 1.1	0.6 ± 1.1	0.4 ± 0.8	0.9 ± 1.3
Margarine	1.3 ± 1.3	1.5 ± 1.3	1.1 ± 1.1	1.6 ± 1.4
Soda and cordials	0.3 ± 0.5	0.2 ± 0.4	0.07 ± 0.2	0.5 ± 0.7
Coffee	2.5 ± 1.1	2.3 ± 1.1	2.1 ± 1.1	2.6 ± 1.0
Energy (kcal)	1432 ± 443	1800 ± 456	1249 ± 330	2000 ± 450
Calcium supplement use (%)^a^	11	23	18	15
Use of multivitaminerals (%)^a^	17	29	25	22
Cohabiting status: living alone (%)^a^	25	23	28	21
Post-menopausal status^a^	91	91	93	90
Smoking status (%)^a^				
Yes	33	18	22	25
No	46	56	53	55
Former	21	26	26	21
≥2 Comorbidities (%)	2.3	2.1	2.4	1.9
Educational level				
≤9 years	85	75	80	80
12 years	6	9	7	7
>12 years	2	7	5	4
Other	7	9	8	8
Physical activity level^a^				
1 (Lowest)	29	15	17	23
2	23	23	22	25
3	30	36	35	32
4	10	13	13	11
5 (Highest)	9	14	13	10
Number of previous fractures	88	62	76	93

^a^Only available in the follow-up 1997 questionnaire. Calcium supplements were taken by 9519 participants and multivitaminerals were taken by 8179 participants. Cohabiting status was reported by 30,925, smoking status by 35,085, physical activity level by 32,314 and post-menopausal status was reported by 35,477 individuals

### Correlations between dietary patterns and nutrients

Observed correlations between dietary patterns identified at each investigation and residual adjusted nutrients are presented in Supplemental Table 2. The Healthy dietary patterns generated at baseline and 1997 were directly correlated with fiber intake as well as with intake of protein and many micronutrients such as vitamins C and E, calcium, magnesium, potassium, while they correlated inversely to saturated fatty acids and sucrose. Most of the observed correlations between the Western/convenience pattern and nutrients were inverse, while sucrose and saturated fatty acids were directly correlated with the pattern. The time-updated dietary patterns correlated with nutrients estimated at baseline and 1997 similarly as dietary patterns formed at each investigation (data not shown).

### Dietary patterns and fracture rate

#### Main analyses

During a median of 25.5 years of follow-up and 1,287,705 person-years at risk, 4997 women experienced a hip fracture. The age and multivariable hazard ratios (HR) (95% CI) of hip fracture are presented in Table 3. In the highest compared to the lowest quartile of the Healthy dietary pattern the multivariable (Model I) adjusted hazard ratio (HR) (95% CI) of a hip fracture was 0.69 (0.64; 0.75). The relationship was approximately linear and the hazard decrease for every additional quartile of the healthy dietary pattern was 11(95% CI 13–9) %. Adding energy intake, BMI and smoking status (Model II) to the model lowered the HR even further [HR 0.57, 95% CI (0.52; 0.62)]. In the highest compared with the lowest quartile of the Western/convenience pattern the multivariable adjusted HR of hip fracture was 1.50 (1.38; 1.62) and was attenuated in Model II [1.22 (1.10; 1.34)]. The linear trend by quartile of the Western/convenience pattern and hip fractures yielded an HR of 1.13 (1.10; 1.16). These relations were independent the other identified pattern (data not shown) and adding fermented milk or milk to Model I resulted in HR (95% CI) 0.54 (0.50; 0.59) and 1.30 (1.20; 1.41) comparing quartile IV with quartile I of the healthy and Western/convenience pattern, respectively.

**Table 3:** Age and multivariable adjusted hazard ratio (HR) and 95% confidence intervals (CI) of hip fracture in quartiles of the time-updated healthy and Western/convenience dietary patterns

	QI	QII	QIII	QIV	Trend per quartile
*Dietary pattern*					
Healthy					
Number of fractures	1501	1304	1190	1002	
Person-years at risk	310,231	321,404	326,650	329,419	
Rate per 1000 person years (95% CI)	4.8 (4.6; 5.1)	4.0 (3.8; 4.3)	3.6 (3.4; 3.9)	3.0 (2.9; 3.2)	
Age-adjusted HR (95% CI)	1.0 (Reference)	0.85 (0.79; 0.91)	0.80 (0.74; 0.86)	0.68 (0.63; 0.74)	0.89 (0.86; 0.91)
Adjusted HR (95% CI) Model I	1.0 (Reference)	0.84 (0.78; 0.91)	0.79 (0.73; 0.86)	0.69 (0.64; 0.75)	0.89 (0.87; 0.91)
Adjusted HR (95% CI) Model II	1.0 (Reference)	0.79 (0.73; 0.85)	0.71 (0.66; 0.77)	0.57 (0.52; 0.62)	0.84 (0.81; 0.86)
Western/convenience					
Number of fractures	1066	1229	1288	1414	
Person-years at risk	319,171	323,351	324,888	320,289	
Rate per 1000 person years (95% CI)	3.3 (3.1; 3.5)	3.8 (3.6; 4.0)	4.0 (3.8; 4.2)	4.4 (4.2; 4.7)	
Age-adjusted HR (95% CI)	1.0 (Reference)	1.26 (1.16; 1.37)	1.34 (1.24; 1.45)	1.48 (1.37; 1.60)	1.13 (1.10; 1.16)
Adjusted HR (95% CI) Model I	1.0 (Reference)	1.24 (1.15; 1.35)	1.32 (1.21; 1.43)	1.50 (1.38; 1.62)	1.13 (1.10; 1.16)
Adjusted HR (95% CI) Model II	1.0 (Reference)	1.17 (1.07; 1.27)	1.17 (1.07; 1.27)	1.22 (1.10; 1.34)	1.06 (1.03; 1.09)

Hazard ratios (95% CI) were determined in Cox proportional hazard analysis. The adjusted models included I) height (continuous), educational level (≤9, 12, >12 years, other), living alone (yes or no), calcium-supplement (yes or no), multivitamineral-use (yes or no), physical activity (5 levels), previous fractures (yes or no), postmenopausal-status (yes or no) and Charlson’s comorbidity index (continuous; 1–16). II) Model I +Total energy, body mass index (both continuous) and smoking status (yes, no and former

*HR* hazard ratio, *CI* confidence interval

#### Sensitivity analysis

**Fig. 2:**
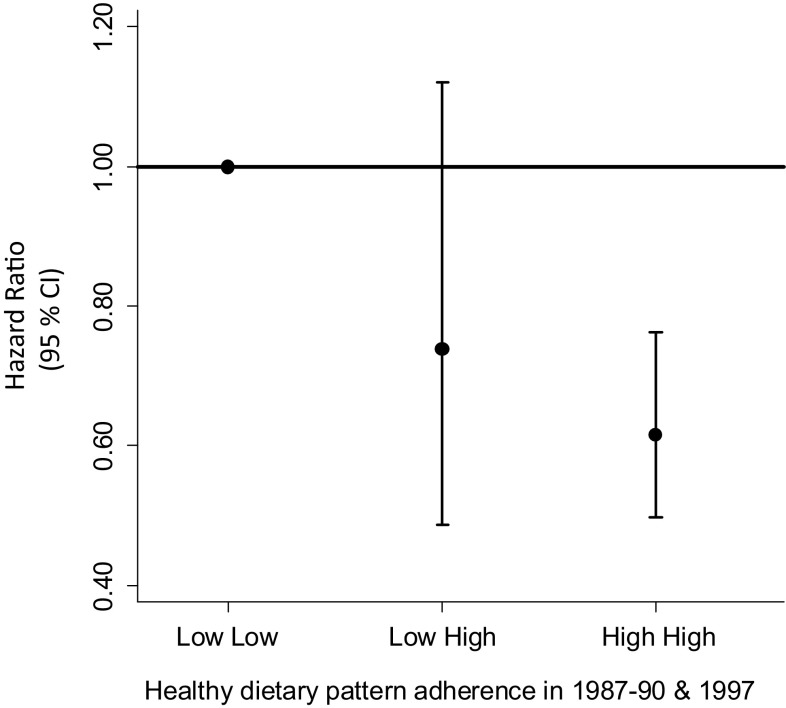
Multivariable adjusted hazard ratio and 95% CI of hip fractures, estimated in Cox proportional hazards regression analysis, comparing the Low Low with the Low High and High High adherence groups of the Healthy dietary pattern. The model was adjusted for height, educational level, living alone, use of calcium-supplements, multivitamineral-use, physical activity, previous fractures, menopausal status and Charlson’s comorbidity index. *Low Low* low adherence both 1987 and 1997, *Low High* change from low to high adherence between investigations, *High High* high adherence both 1987 and 1997

**Fig. 3:**
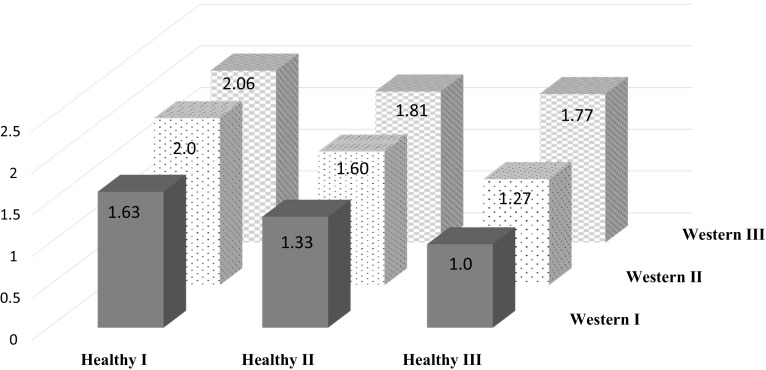
Multivariable adjusted hazard ratio (HR) of hip fractures in joint classified participants across nine strata formed with the tertiles of the Healthy and the Western/convenience dietary pattern. The Healthy III and Western/convenience I was used as the reference (HR = 1.0) category. *P* for all ≤0.001. The model was adjusted for height, educational level, living alone, use of calcium-supplements, multivitamineral-use, physical activity, previous fractures, menopausal status and Charlson’s comorbidity index. The tertiles of respective dietary pattern were named as follows. Heathy I, Healthy II and Healthy III and Western/convenience I, Western/convenience II and Western/convenience III

Complete case analysis (n = 27, 238, including 2163 hip fracture cases) confirmed the results of the study, although with somewhat wider CIs but not attenuated estimates. The multivariable adjusted HR (95% CI) (Model I) in the ascending quartiles were 0.85 (0.76; 0.96), 0.80 (0.71; 0.90), 0.59 (0.52; 0.67) for the time-updated Healthy pattern and 1.54 (1.35; 1.77), 1.84 (1.62; 2.11), 2.12 (1.86; 2.42) for the time-updated Western/convenience pattern. Additional adjustment in the complete case analysis for history of falls did not change the points estimates. If the dietary patterns retained from each investigation were analysed separately (without a time-updated analysis), the estimates were materially not different from the main analysis although weaker − the multivariable adjusted HR (95% CI) (Model I) of a first hip fracture in the highest compared with the lowst quartile was 0.78 (0.72; 0.85) and 1.12 (1.04; 1.21) for the Healthy and Western/convenience patterns in 1987, respectively. The corresponding multivariable adjusted HR (95% CI) using the 1997 data was 0.73 (0.65; 0.82) for the Healthy pattern and 1.21 (1.07; 1.36) for the Western/convenience dietary pattern. Compared to a low adherence, high adherence to a healthy dietary pattern at both investigations was inversley related to the risk of hip fracture (Fig. 2). Change of eating behavior between investigations, from low to high adherence of a Healthy pattern, was associated with lower risk of hip fracture although not statistically significant. In joint classification of study participants according to adherence to the different dietary patterns, the risk of hip fracture was higher with higher adherence to the Western/convenience pattern in each stratum of the Healthy dietary pattern (Fig. 3). Thus, the more healthy the overall diet was, the lower the rate of hip fractures.

## Discussion

The present study indicates that women following a healthier dietary pattern consisting of more vegetables, fruits and cereals, fish and fermented milk have 31% lower hip fracture rate (highest vs. lowest quartile). In contrast, women following a more Western/convenience pattern, consisting of sweet snacks and bakery products, jams, sodas, savoury snacks and white bread, had a 50% higher rate of hip fracture in the highest versus the lowest quartile. The analysis with joint classified participants revealed an unfavourable effect of the Western/convenience pattern on hip fracture rate in each stratum of a Healthy dietary pattern (Fig. 3). This indicates that the inverse association of the Healthy dietary pattern may be reduced by a more Western/convenience dietary pattern. Further, women who were highly adherent to the Healthy dietary pattern at both investigations had the lowest hip fracture rate. Those changing from a low to high adherence between investigations exhibited lower, albeit not statistically significant, hip fracture rate than those who remained in the lowest quartile of adherence.

That a healthy diet high in vegetables, fruits, nuts, cereals, fish, low fat dairy and vegetable oil, but low in meats, refined grains and sugars may reduce the risk of chronic metabolic diseases [13, 35] is established and recognized in current dietary guidelines [36, 37]. Mechanisms that link healthy dietary components also with fewer fractures may relate to the supply of nutrients and other bioactive compounds needed to maintain healthy bone tissues and supportive muscles [4, 38]. The supply of adequate nutrients may positively affect bone remodelling and bone mineral density (BMD) [2, 4], although this has not been observed in all studies [20, 39]. Inflammation and sequential or concomitant oxidative stress are linked to osteoporosis, lower BMD and has been implicated in age-related bone loss [40–42] and sarcopenia. Sarcopenia, characterized by a decline in muscle mass and strength, is linked to risk of falls and thereby fractures [43]. A diet with an adequate intake of antioxidants, such as vitamins E and C and scavengers of free radicals [44, 45], may therefore lessen the risk of fractures [46], as may n-3 fatty acids found in fish and vegetable oils [47]. Moreover, cardiovascular disease [48] and type 2 diabetes [49] are associated with risk of fractures. Thus, prevention of cardiovascular disease and type 2 diabetes may indirectly prevent fractures. Another modifiable lifestyle factor important for bone health is regular physical activity [50], but the results of the present study were independent of physical activity. Accounting for the history of falls did not have an influence on the results.

Healthy dietary patterns generated by PCA or other data reduction techniques are fairly stable across populations and have in meta-analyses been inversely related to the development of cardiovascular disease [51], type 2 diabetes [52], certain cancers [13] and other diseases [53]. However, the evidence for data derived patterns and risk of fracture is less certain. In a study of men (Health Professional Follow-up) and women (Nurses’ Health Study; NHS), no association between dietary patterns, neither healthy nor western, and risk of hip fractures was observed [16]. Despite continuous updates of exposures and confounders, the lack of association may be because of a more limited exposure width or that fracture events were self-reported, which may have biased estimates given the high mortality rates after a hip fracture event. Indeed, the hip fracture rate in the NHS was 1 per 1000 person-years (lower than should be expected in an American population [54]) compared with 4 per 1000 person-years in the SMC although the women were comparable in terms of age, BMI and follow-up.

The observation of an inverse relation between a Healthy dietary pattern and incident hip fracture rates in the present study is partly supported by previous studies examining healthy diet scores, for example the Mediterranean diet score, and hip fractures [55]. A positive association between healthy dietary patterns and BMD [56–58] has also been reported. The Healthy dietary pattern in the present study was positively correlated with many nutrients of importance for bone (e.g. vitamin E and C, calcium, potassium, magnesium and protein) [38]. In line with this, a nutrient dense dietary pattern was associated with a decreased risk of low-trauma fractures in postmenopausal women, while an energy dense pattern (soda, potato chips, French fries, certain meats and desserts) was not related to fracture risk [17]. In the present study the Western/convenience pattern was related to a higher rate of hip fractures and correlated positively with intake of sugar and fat, while negatively to micronutrients. That we were able to detect a positive relation may be due both study size and wide exposure range. A Western type of dietary pattern has been positively related to inflammatory markers [59] and to lower BMD [60], both associated with fracture risk. Further, a Western diet is relatively nutrient poor and may displace healthy foods and thus important nutrients and bioactive components needed for bones and supportive muscles, as shown in our analysis with jointly classified participants (Fig. 3). Individual components of a Western diet, such as sugars [61] and saturated fatty acids [62] per se, could have detrimental effects on bone although results are inconclusive. A recent meta-analysis reported that a Western dietary pattern was consistently associated with an increased risk of type 2 diabetes [52] while the association to cardiovascular disease was less certain [51], further emphasizing a healthy dietary pattern to improve health. Adjusting the analysis with the Western/convenience pattern for milk intake attenuated the HR of hip fracture from 1.50 to 1.30, indicating that milk may be a potential mediator of the association. This accords with the results in our previous study [8], while not with all studies [63].

This present population-based cohort study includes a greater number of hip fractures than any other single cohort study in the world. Further strengths include the complete ascertainment of hip fractures from a nationwide patient register with no loss to follow-up and the repeated assessment of diet and a large number of potential covariates. Although we adjusted for important covariates as proxy for socioeconomic status, there may be an influence of residual or unmeasured confounding. However, such residual confounder effect needs to be quite strongly associated with exposure or outcome (RR ~ 2.30) [64]. This is unlikely given that known socioeconomic factors, such as education level, are only weakly associated with hip fractures [65]. Although diet was assessed twice which is a strength rendering improved validity of the dietary assessment, there is a possibilty that dietary changes by time also affect the construct and comparison of different dietary scores by PCA. This seems to be a minor limitation given that similar results were obtained by use of time-updated information and by use of single exposure assessments. Nonetheless, the collection of diet data is inherently prone to a number of limitations, affecting both the precision and accuracy of the measurement and leading to conservatively biased estimates. However, the study questionnaires has the ability to rank participants and have been found valid and reproducible [66, 67] and the large study size will compensate at least partly for random misclassification. In addition, our results might not apply to other ethnicities or men.

In conclusion, a higher adherence to a Healthy dietary pattern characterized by high quality foods including plant foods, grain cereals, fish and fermented milk was associated with a lower rate, while higher adherence to a Western/convenience dietary pattern was associated with a higher rate of hip fractures in women. These results harmonize with studies investigating dietary patterns and risk of chronic metabolic diseases and suggest that eating a varied healthy diet is beneficial for the prevention of fragility fractures in women.

## Electronic supplementary material

Below is the link to the electronic supplementary material.

Supplementary material 1 (DOCX 18 kb)

## Abbreviations

FFQ: Food frequency questionnaire
PCA: Principal component analysis
BMD: Bone mineral density
SMC: Swedish Mammography Cohort
DAG: Directed acyclic graphs
