# Supplementary material for: Long-term a posteriori dietary patterns and risk of hip fractures in a cohort of women
Source: Eur J Epidemiol. 2017 Jun 5;32(7):605–16. doi: 10.1007/s10654-017-0267-6 (PMC5570766; doi:10.1007/s10654-017-0267-6)
Supplement: Supplementary file 1 — Supplementary material 1 (DOCX 18 kb) [file 10654_2017_267_MOESM1_ESM.docx]

**Supplemental Table**

**Supplemental Table 1.** Age and multivariable adjusted Hazard Ratio (HR) and 95 % Confidence Intervals (CI) of hip fracture in quartiles of the time-updated alcohol & snack pattern.

| Alcohol & Snack |  |  |  |  |  |
| --- | --- | --- | --- | --- | --- |
|  | QI | QII | QIII | QIV | Trend per quartile |
| Number of fractures | 1801 | 1463 | 997 | 736 |  |
| Person-years at risk | 302,476 | 316,401 | 329,767 | 339,060 |  |
| Rate per 1000 person years (95% CI) | 6.0 (5.7; 6.2) | 4.6 (4.4 ; 4.9) | 3.0 (2.8 ; 3.2) | 2.2 (2.0 ; 2.3) |  |
| Age-adjusted HR (95% CI) | 1.0 (Reference) | 1.00 (0.93; 1.07) | 0.87 (0.80; 0.94) | 0.87 (0.80; 0.95) | 0.95 (0.92; 0.97) |
| Adjusted HR (95% CI) ^Model I^ | 1.0 (Reference) | 1.00 (0.92; 1.06) | 0.87 (0.81; 0.94) | 0.93 (0.85; 1.01) | 0.96 (0.93; 0.99) |
| Adjusted HR (95% CI) ^Model II^ | 1.0 (Reference) | 1.07 (1.00; 1.15) | 0.96 (0.88; 1.04) | 0.99 (0.90; 1.09) | 0.99 (0.96; 1.02) |
|  |  |  |  |  |  |

HR hazard ratio, CI confidence interval

Hazard ratios (95% CI) were determined in Cox Proportional hazard analysis.

The adjusted models included I) height (continuous), educational level (<=9, 12, >12 years, other), living alone (yes or no), calcium-supplement (yes or no), multivitamineral-use (yes or no), physical activity (5 levels), previous fractures (yes or no), postmenopausal-status (yes or no) and Charlson’s comorbidity index (continuous; 1-16). II) Model I +Total energy, body mass index (both continuous) and smoking status (yes, no and former)

**Supplemental table 2.** Spearman rank correlations between the Healthy and Western/Convenience patterns and intake of residual adjusted nutrients per day and energy at baseline 1987-90.

|  | Baseline 1987-90 | | 1997 | |
| --- | --- | --- | --- | --- |
|  | Healthy | Western/  convenience | Healthy | Western/  convenience |
| Protein (g) | 0.34 | -0.05 | 0.20 | -0.28 |
| Carbohydrate (g) | 0.06 | -0.19 | 0.17 | 0.12 |
| Fiber (g) | 0.29 | -0.43 | 0.49 | -0.25 |
| Sucrose (g) | -0.08 | 0.25 | -0.12 | 0.28 |
| Saturated fatty acids (g) | -0.25 | 0.15 | -0.24 | 0.16 |
| Monounsaturated fatty acids (g) | -0.28 | 0.29 | -0.36 | 0.14 |
| Polyunsaturated fatty acids (g) | -0.19 | 0.05 | -0.04 | -0.14 |
| n-6 fatty acids (g) | -0.30 | ns | -0.04 | -0.11 |
| n-3 fatty acids (g) | 0.42 | 0.01 | 0.03 | -0.27 |
| Vitamin C (mg) | 0.48 | -0.14 | 0.34 | -0.33 |
| Vitamin E (mg) | 0.34 | -0.13 | 0.31 | -0.33 |
| Retinol (mg) | 0.09 | 0.10 | -0.11 | 0.06 |
| Vitamin D (µg) | 0.13 | -0.06 | -0.03 | -0.04 |
| Calcium (mg) | 0.15 | -0.13 | 0.16 | -0.03 |
| Iron (mg) | -0.04 | 0.03 | 0.23 | -0.32 |
| Folate (µg) | 0.63 | -0.37 | 0.46 | -0.38 |
| Potassium (mg) | 0.54 | -0.25 | 0.45 | -0.27 |
| Magnesium (mg) | 0.48 | -0.38 | 0.51 | -0.29 |
| Sodium (mg) | 0.31 | 0.05 | 0.05 | -0.28 |
| Phosphorous (mg) | 0.30 | -0.24 | 0.32 | -0.17 |
| Energy intake (kcal) | 0.33 | 0.66 | 0.57 | 0.61 |

All p <0.001

ns not significant
